# Supplementary material for: Surface Complex V couples proton gradient across the plasma membrane to ATP production in cancer
Source: Res Sq. 2026 Jun 17:rs.3.rs-10007862. Preprint. [Version 1] doi: 10.21203/rs.3.rs-10007862/v1 (PMC13308370; doi:10.21203/rs.3.rs-10007862/v1)
Supplement: 1 [file NIHPPRS10007862V1-supplement-1.pdf]

Supplementary Figure Legends

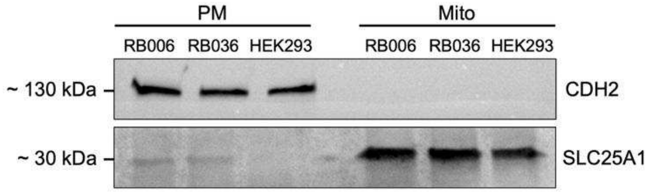

Supplementary Fig. 1 | Validation of plasma membrane fraction purity. Immunoblot analysis of isolated plasma membrane and mitochondrial fractions from RB006, RB036 and HEK293 cells probed for the plasma membrane marker CDH2 and mitochondrial marker SLC25A1. Representative of three biologically independent experiments.

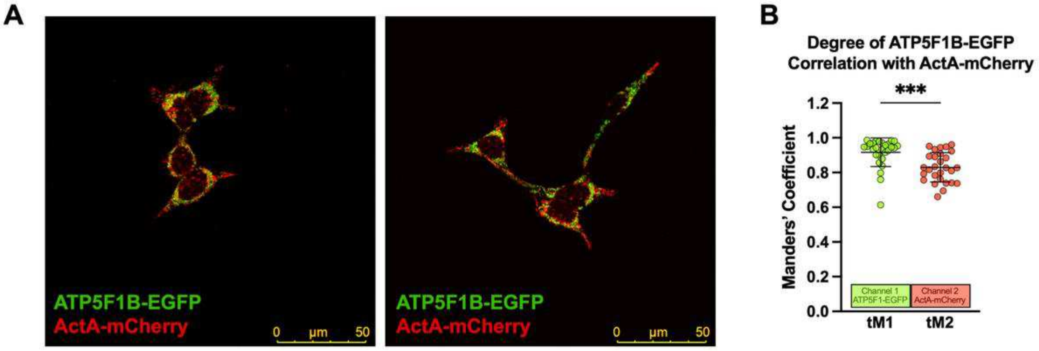

Supplementary Fig. 2 | ATP synthase and mitochondrial localization in HEK293 cells.
a Validation of ActA-mCherry . a, Representative confocal images of HEK293 cells expressing ATP5F1B-EGFP and mitochondria-targeted ActA-mCherry. **b,** Quantification of ATP5F1B-EGFP colocalization with mitochondria-targeted ActA-mCherry by Manders' coefficients (tM1 and tM2). Data are from three biologically independent experiments. *** $P < 0.001$. Statistics: two-tailed Welch's ***t***-test (b). Error bars: mean ± s.d.

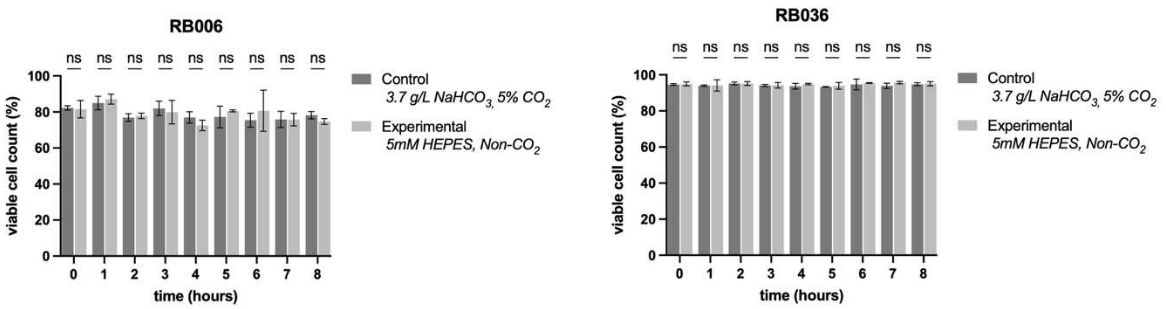

Supplementary Fig. 3 | Control for culturing in HEPES-buffered media with non-CO_2 incubation. Time-course measurement of cellular viability, measured with Trypan Blue exclusion assay, in cells cultured under normal (3.7 g/L $NaHCO_3$ w/ CO_2 incubation) or experimental (5mM HEPES w/ non-CO_2 incubation) conditions. Statistics: one-way ANOVA with Tukey's multiple comparisons test. Error bars: mean ± s.d.
